# Bigger is better: changes in body size explain a maternal effect of food on offspring disease resistance

**DOI:** 10.1002/ece3.2709

**Published:** 2017-02-03

**Authors:** Jennie S. Garbutt, Tom J. Little

**Affiliations:** ^1^ Ashworth LaboratoriesInstitute of Evolutionary BiologyThe University of EdinburghEdinburghUK

**Keywords:** host–parasite, life‐history, maternal effects, mechanism, trans‐generational effects

## Abstract

Maternal effects triggered by changes in the environment (e.g., nutrition or crowding) can influence the outcome of offspring–parasite interactions, with fitness consequences for the host and parasite. Outside of the classic example of antibody transfer in vertebrates, proximate mechanisms have been little studied, and thus, the adaptive significance of maternal effects on infection is not well resolved. We sought to determine why food‐stressed mothers give birth to offspring that show a low rate of infection when the crustacean *Daphnia magna* is exposed to an orally infective bacterial pathogen. These more‐resistant offspring are also larger at birth and feed at a lower rate. Thus, reduced disease resistance could result from slow‐feeding offspring ingesting fewer bacterial spores or because their larger size allows for greater immune investment. To distinguish between these theories, we performed an experiment in which we measured body size, feeding rate, and susceptibility, and were able to show that body size is the primary mechanism causing altered susceptibility: Larger *Daphnia* were less likely to become infected. Contrary to our predictions, there was also a trend that fast‐feeding *Daphnia* were *less* likely to become infected. Thus, our results explain how a maternal environmental effect can alter offspring disease resistance (though body size), and highlight the potential complexity of relationship between feeding rate and susceptibility in a host that encounters a parasite whilst feeding.

## 1 Introduction

Maternal effects occur when the phenotype of an individual is determined, in part, by the conditions experienced by its mother and her phenotype, irrespective of the genes transmitted from mother to offspring (Cheverud & Moore, 1994; Kirkpatrick & Lande, 1989; Mousseau & Fox, 1998a, 1998b; Wolf, Brodie, Cheverud, Moore, & Wade, 1998; Wolf & Wade, 2009). Maternal effects are increasingly recognized to profoundly affect the expression of infectious disease in vertebrates (Brinkhof, Heeb, Kölliker, & Richner, 1999; Gasparini et al., 2007; Klasing, 1998; Tella, Bortolotti, Dawson, & Forero, 2000), invertebrates (Boots & Roberts, 2012; Gibbs, Breuker, Hesketh, Hails, & Van Dyck, 2010; Huang & Song, 1999; Little, O'Connor, Colegrave, Watt, & Read, 2003; Lorenz & Koella, 2011; Ma et al., 2005; Miller, Pell, & Simpson, 2009; Mitchell & Read, 2005; Rahman, Roberts, Sarjan, Asgari, & Schmidt, 2004; Roth et al., 2010; Stjernman & Little, 2011; Tidbury, Pedersen, & Boots, 2011), and plants (Grünzweig, 2011; Holeski, Jander, & Agrawal, 2012). Because of their distinct evolutionary features (Kirkpatrick & Lande, 1989; Mousseau & Fox, 1998b; Wade, 1998), maternal effects are likely to affect the evolution of hosts and their parasites in complex and difficult‐to‐predict ways that have not yet been fully explored.

The paradigmatic example of a maternal effect on disease resistance is the transfer of immunity via antibodies from mother to offspring in vertebrates (Hasselquist & Nilsson, 2009). However, many organisms, and especially invertebrates, are well known to show maternal effects on resistance when mothers experience environmental variation, for example, temperature or food variation (Garbutt, Scholefield, Vale, & Little, 2014; Mitchell & Read, 2005; Triggs & Knell, 2012). Both the mechanisms and adaptive significance of such maternal environmental effects on resistance in invertebrates are unclear. Mothers might use environmental conditions as cues for disease risk and change offspring resistance accordingly. This might be achieved through the transfer of immune molecules or by altering offspring life history in a manner that specifically improves resistance. However, changed resistance could also come about as a side effect of life‐history changes that are specific adaptations to the environment the mother has experienced. For example, mothers kept under harsh conditions may produce offspring with certain behavioral or life‐history phenotypes that are well suited to harsh environments, but that also lead to a changed encounter, and ultimately infection, rate with pathogens. Maternal effects on disease mediated through changes in life history are unlikely to be taxonomically restricted, and thus, such maternal effects might represent a neglected source of variation.

Here, we investigate the mechanism underlying a maternal effect of food on disease resistance in *Daphnia magna*. In this system, mothers held in poor nutritional conditions produce fewer offspring that are more resistant (their probability of becoming infected is lower) to *Pasteuria ramosa,* a bacterial parasite (Ben‐Ami, Ebert, & Regoes, 2010; Mitchell & Read, 2005; Stjernman & Little, 2011). The offspring of poorly fed mothers are also larger at birth (Garbutt et al., 2014; Guinnee, Gardner, Howard, West, & Little, 2007; Guinnee, West, & Little, 2004; Stjernman & Little, 2011) and feed at a lower rate than the offspring of well‐fed mothers (Garbutt & Little, 2014). Here, we generate plausible hypotheses linking these correlated life‐history traits to changes in susceptibility, and test which is causal by conducting a large experiment in which we measure susceptibility and life‐history traits in individual *Daphnia*.

Our first hypothesis concerns feeding rate: Because the offspring of low‐food mothers have a reduced feeding rate, and because *P. ramosa* infects via the gut (Duneau, Luijckx, Ben‐Ami, Laforsch, & Ebert, 2011; Ebert et al., 1996), we propose that the lower rate of infection suffered by these *Daphnia* arises because they ingest fewer spores. Food quantity and quality has been linked with the ability of *Daphnia dentifera* hosts to resist the fungal pathogen, *Metschnikowia bicuspidata* (Hall et al., 2009). Our second hypothesis is that offspring quality plays an important role and in particular that protection is conferred by the improved general provisioning of the offspring of low‐food mothers. Because *Daphnia* generally obey a trade‐off between offspring size and number (Guinnee et al., 2004, 2007; Smith & Fretwell, 1974), it is expected that large individuals are better provisioned and thus perhaps better at defending themselves against parasites. These two hypotheses highlight the delicate balance for hosts that encounter their parasites whilst feeding: Such hosts need to obtain sufficient nutrients for defense (as well as growth and maintenance), but risk infection whilst feeding through the uptake of environmental spores. This trade‐off is certainly not restricted to *Daphnia* species, as a diverse range of hosts also encounter their parasites whilst feeding or foraging (Fenton, Fairbairn, Norman, & Hudson, 2002; Williams & Barker, 2001; Wobeser, 2005).

To test these competing hypotheses, we manipulated maternal food availability and then measured body size, feeding rate, and susceptibility in each individual offspring to disentangle which factor is most tightly linked with susceptibility. To achieve the power necessary to disentangle these effects, we performed the experiment using a single clone of *Daphnia,* thus minimizing any variation in susceptibility arising from genetic differences.

## 2 Methods

### 2.1 Organisms

The pathogen *P. ramosa* is a spore‐forming bacterium whose main fitness effect is to cause sterilization in hosts (Ebert et al., 1996). The host *D. magna* (Crustacea: Cladocera) is a planktonic crustacean commonly found in small freshwater ponds. In this study, we used clone Kc49a, a genotype from the Kaimes pond near Leitholm in the Scottish Borders. A previous study of 24 genotypes from this population (Stjernman & Little, 2011) demonstrated, despite substantial genetic variation, that the average effect is for lower infection levels after maternal food restriction. We specifically focused the current experiments on clone Kc49a because this clone exhibits the phenotype we know to be typical of this population; that is, that low maternal food raises the resistance of offspring (Stjernman & Little, 2011). By removing genetic effects from the equation, a single‐clone experiment offers a simplified, powerful test of what is possible in this system (see Little & Colegrave, 2016 for discussion), and because we have chosen a clone that shows the typical response of all genotypes in this population, our experiment reveals what is probable for this population.

The *P. ramosa* isolate we used (called Kaimes 1) was isolated from sediment samples in the same location. Horizontal transmission of *P. ramosa* is achieved when spores are released from dead hosts and picked up by filter feeding *Daphnia* (Ebert et al., 1996). Vertical transmission has never been observed. Infections are easy to diagnose with the naked eye: *Daphnia* have a clear carapace, and reddish‐brown bacterial growth is visible in the hemolymph.

### 2.2 Acclimation

In this experiment, mothers (the F_o_ generation) were raised under either high or low food and body size, feeding rate, and parasite susceptibility were measured in their offspring (the F_1_ generation). Initially, 180 replicates, each an individual *Daphnia* in a 60‐ml media‐filled glass jar, were acclimatized for three generations under standardized conditions at a light:dark cycle of 12:12 L:D in controlled climate chambers at 20°C. *Daphnia* were kept in synthetic pond medium (Klüttgen, Dülmer, Engels, & Ratte, 1994) and were fed on *Chlorella* spp, a green algae cultured in chemostats with Chu B medium. Food quantity during this period was 1 density unit/jar/day (one density unit is the optical density of 650 nm white light by the *Chlorella* culture, which represents about 5 × 10^6^ algal cells). Media was changed when offspring were observed in the jar, or, if none were present, every third day. Acclimating all replicates for three generations is a process designed to equilibrate uncontrolled maternal effects and ensure that each replicate is independent (Ebert, Zschokke‐Rohringer, & Carius, 1998).

### 2.3 Maternal (F_0_) generation

From the second clutch of the third acclimatizing generation, we took two offspring from each replicate and assigned them to two maternal (F_0_) food treatments (high food—1.0 density units/jar/day and low food—0.3 density units/jar/day). Thus, at this stage of the experiment, there were 360 jars. Media was changed twice a week and when offspring were present. From the second clutch of the maternal (F_0_) generation, we took one offspring from each replicate jar to set up the (F_1_) offspring.

### 2.4 Offspring (F_1_) generation

We measured the body size, feeding rate, and susceptibility of each F_1_ *Daphnia*. For body size measurements, *Daphnia* were photographed on their day of birth with an Olympus D20 digital camera attached to a stereoscope. These pictures were later used for measurement of body length, which was taken from the center of the eye to the base of the tail spine in ImageJ v1.46r (http://rsbweb.nih.gov/ij/) in pixels and subsequently translated into millimeters.

Immediately following photography, we measured the feeding rate of each *Daphnia* by determining how quickly they filter algae from the water column based on changes in optical density as described in Garbutt and Little (2014). For this, the *Daphnia* were placed individually in the well of a 24‐well plate (Costar Corning, NY, USA). Excess media was removed and 2‐ml media containing 1.0 density units *Chlorella* algae added to each well. Six control wells per plate did not contain any *Daphnia*. The plates were incubated for 24 hr (so from day 0 to day 1) at a light/dark cycle of 12:12 L:D in controlled climate chambers at 20°C. Following this incubation period, the contents of each well were mixed by pipetting and three aliquots of 200 μl removed to the wells of a 96‐well plate (Costar Corning, NY, USA). The optical density of 650 nm white light by each well was determined using a plate‐reading spectrophotometer (BioTek) and the mean calculated for the three replicate wells. Clearance rate (feeding rate) for each *Daphnia* was calculated by subtracting this mean value from the mean optical density of the six plate controls.

Pathogen exposure occurred immediately after the measurement of feeding rate (and therefore exposures started on day 1). *Daphnia* were removed from the feeding rate assay and placed individually in jars with sand and inoculated with 50,000 *P. ramosa* transmission spores per jar. *Daphnia* were exposed for 7 days: During this period, media was not changed and individuals were fed daily 1.0 density units/jar. At the end of the 7‐day exposure period, *Daphnia* were transferred into new jars with fresh media; for the remainder of the experiment, media was changed every third day and when offspring were present. The feeding regime remained the same (i.e., 1.0 density units/jar daily). We observed the F_1_ *Daphnia* until day 37, at which point infections could be confirmed visually by observing the symptoms of *P. ramosa* infection (lack of eggs in the brood chamber and reddish color). At the end of the 37‐day observation period, we recorded whether each host was infected or not.

### 2.5 Analysis

We first constructed simple models with maternal food as the sole explanatory variable to test the effect of food treatment on feeding rate, body size, and the likelihood of becoming infected. Feeding rate and body size are continuous variables and were analyzed in a linear model. Infection status is a binary response variable and was analyzed in a generalized linear model (link = logit, dist = binary). These analyses were performed in JMP^®^ Version 10.00 (SAS Institute Inc.) Cary, NC, 1989‐2007.

Next we used path analysis to examine the relationship(s) between maternal food, feeding rate, body size, and the probability of becoming infected. Initially, a full model was fitted including all possible relationships between all four variables, and this was simplified by removing the least significant term until only significant paths remained. Initially, we did not include a path from feeding rate to body size because we measured body size at birth and feeding rate the day after, so feeding rate could not influence body size. Because infection status and maternal food were binary variables (infected or not; high or low food), we recoded them as ordinal variables (0/1) and specified infection status (which, unlike maternal food, is a response or “endogenous” variable) as an ordered variable using the “ordered” function.

All path analyses were conducted using the “lavaan” package (SEM function) in R (Rosseel, 2012). Model fit was indicated by a Comparative Fit Index (CFI), and the strength of each path was assessed by comparing standardized path coefficients. Higher absolute values of path coefficients indicate a more parsimonious path, and indirect paths were calculated by multiplying the coefficients. When there was more than one significant path between two variables, the net effect was calculated by summing the path coefficients of all paths. Because the models contain categorical variables, care must be taken in interpreting the direction of relationships from the path coefficients. Our coding of these variables means that a positive coefficient results if low maternal food positively affects a continuous variable and if a continuous variable increases the probability of becoming infected.

To plot infection risk against body size and feeding rate, we used generalized linear models (link = logit, dist = binary) with body size and feeding rate as explanatory variables, and plotted the values predicted by the model. We also analyzed feeding rate in a linear model with body size as the sole explanatory variable to obtain an estimate of the strength of the relationship between these two continuous variables (*R* ^2^).

## 3 Results

**Figure 1:**
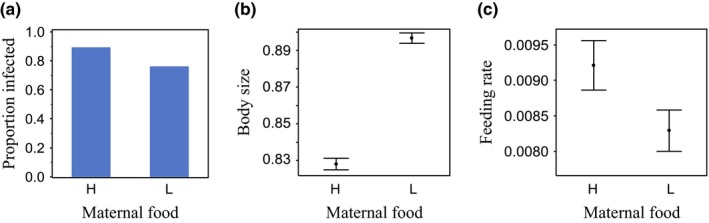
Maternal food and offspring phenotype. Maternal food (high food—H; low food—L) affects offspring (a) disease resistance (proportion of *Daphnia* that became infected with *Pasteuria ramosa),* (b) body size at birth (mean ± *SE*), and (c) feeding rate (mean ± *SE*)

Maternal food influenced body size at birth, feeding rate, and the probability of becoming infected when each was analyzed separately (Table 1, Figure 1): The offspring of food‐restricted mothers were less likely to become infected, were larger at birth, and had a lower feeding rate than the offspring of well‐fed mothers.

**Table 1:** Output of simple models with maternal food as the sole explanatory variable. Results from general linear models (body size and feeding rate; *F*‐test statistic) and generalized linear model (probability of infection; χ^2^ test statistic)

Response	Effect	*df* (effect, error)	*F*/χ^2^	*p*
Probability of becoming infected	Maternal food	1	10.45	.0012
Body size	Maternal food	1, 332	260.54	<.0001
Feeding rate	Maternal food	1, 332	4.09	.044

**Figure 2:**
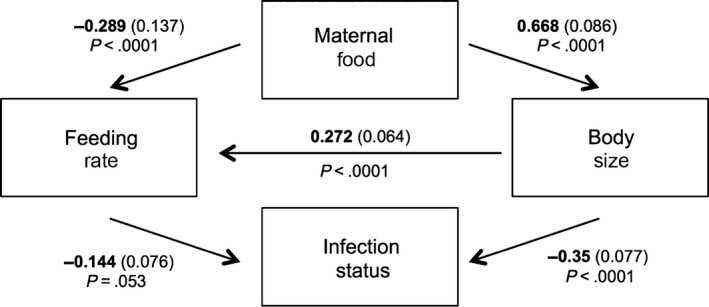
Path analysis of routes linking maternal food with the probability of becoming infected following exposure to *Pasteuria ramosa*. Minimal path model with path coefficients, standard error (in brackets), and *p*‐values shown next to each significant path

**Figure 3:**
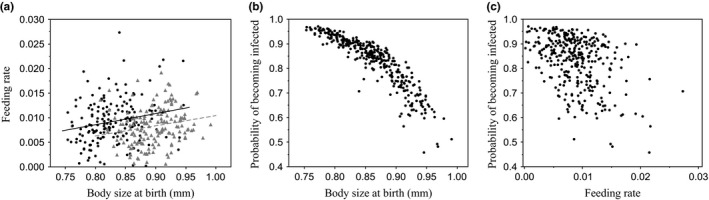
Relationship between body size, feeding rate, and the probability of becoming infected. (a) Feeding rate and body size in the offspring of high‐food (black circles, black line) and low‐food (gray triangles, dashed line) mothers. (b, c) Probability of becoming infected as predicted by a general linear model with body size and feeding rate as explanatory variables

The only insignificant path removed from the path model was that from maternal food to infection status (*p* = .55, path coefficient = −.060, *SE* = 0.210, final CFI: 1.00). Path analysis revealed a slightly complex relationship between maternal food and feeding rate: Maternal food affected feeding rate both directly, with the offspring of low‐food mothers feeding more slowly (Figure 2; Table 2; path coefficient −.289), and indirectly via body size, with the offspring of low‐food mothers being larger and larger individuals feeding more rapidly (Figures 2 and 3a; Table 2; path coefficient .668 × .272 = .182). These opposing effects drive the observed overall effect that the offspring of low‐food mothers feed slowly (see Figure 1c), because the direct effect is stronger than the indirect effect (sum of path coefficients −.289 + .182 = −.107). In other words, the reduction in feeding rate from being born to a low‐food mother is not entirely compensated for by the increase in feeding rate from being born larger. Importantly, the relationship between body size and feeding rate is not tight (Figure 3; linear model of feeding rate, with body size and maternal food as explanatory variables; *R* ^2^ = .047), which means we are able to distinguish between the effects of each variable on infection status.

**Table 2:** Path analysis of potential routes from maternal food to infection status. The standardized path coefficients, the standard error of the coefficient, and the *p* value for each path in the analysis

Path	Coefficient	*SE*	*p*
Maternal food → feeding rate	−.289	0.137	<.0001
Maternal food → body size	.668	0.086	<.0001
Body size → feeding rate	.272	0.064	<.0001
Body size → infection status	−.350	0.077	<.0001
Feeding rate → infection status	−.144	0.076	.053
*Excluded paths*			
Maternal food → infection status	−.060	0.210	.412

Indeed, our primary interest was in identifying the likeliest path from maternal food to infection status. Infection status was affected by body size, with larger individuals being less likely to become infected (Figures 2 and 3b). There was also a trend (*p* = .053) that infection status was affected by feeding rate, with fast‐feeding individuals less likely to become infected (Figures 2 and 3c). Only the path via body size can explain the link between maternal food and feeding rate, as the larger‐at‐birth individuals from low‐food mothers are less likely to become infected (path coefficient .668 × −.350 = −.234). Whilst feeding rate does perhaps affect susceptibility (and we recognize that we may have had less power to detect relationships with feeding rate because estimates of feeding rate were more variable than the estimates of body size), it cannot be responsible for the link between maternal food and feeding rate, because the trend is that the slow‐feeding offspring of low‐food mothers are more likely to become infected.

## 4 Discussion

*Daphnia magna* mothers held in poor nutritional conditions produce offspring that are less likely to become infected with the bacterial parasite, *P. ramosa* (Figure 1a; Garbutt et al., 2014; Mitchell & Read, 2005; Stjernman & Little, 2011). Maternal food also affects offspring size at birth (Figure 1b; Garbutt et al., 2014; Guinnee et al., 2004, 2007; Stjernman & Little, 2011) and feeding rate (Figure 1c; Garbutt & Little, 2014)). Our goal here was to disentangle which of these maternally determined traits is most tightly linked to changes in susceptibility. Maternal food most strongly affects offspring susceptibility to infection via changes in offspring body size (Figures 2 and 3b). Although there was a trend that feeding rate also affected susceptibility, this cannot explain the link between maternal food and susceptibility because it acts in the opposite direction (the trend is that the slow‐feeding offspring of low‐food mothers are more likely to become infected; Figures 2 and 3c).

These findings support the life‐history theory prediction (Godfray, 1987; Lloyd, 1987; Parker & Begon, 1986; Smith & Fretwell, 1974; Wilson & Lessells, 1994) that larger, better‐provisioned offspring generally perform better. Because immune defenses are costly (Moret & Schmid‐Hempel, 2000), larger individuals that have greater access to resources because of their size may be able to launch and sustain stronger defenses. Future experiments can explore whether enhanced immune competence is related to particular maternal provisions [for instance, polyunsaturated fatty acids (Schlotz, Sørensen, & Martin‐Creuzburg, 2012; Wacker & Martin‐Creuzburg, 2007)]. Body size at birth has previously been shown to account for variation in many life‐history traits in *D. magna* (Ebert, 1991); our results now expand this to show that it is also an important determinant of susceptibility. Variation in body size at birth might also explain other environmental maternal effects. In particular, body size might link maternal temperature with offspring disease resistance in *Daphnia*, because the more‐resistant offspring of mothers held at higher temperatures are also larger at birth (Garbutt et al., 2014).

That low maternal food causes altered disease resistance through an increase in offspring body size suggests that this maternal effect is not a specific adaptation to parasite resistance, but instead a general stress response. A key expectation of this theory is that the offspring of low‐food mothers will perform better in a number of stressful environments. There is some evidence that offspring of low‐food mothers (Gliwicz & Guisande, 1992; Gorbi, Moroni, Sei, & Rossi, 2011), and larger offspring (Tessier, Henry, Goulden, & Durand, 1983), are more starvation resistant, but further experiments are needed to characterize the stress resistance of the offspring of food‐restricted mothers.

Our results shed light on the relationship between feeding rate and susceptibility. Contrary to our expectation that fast‐feeding *Daphnia* should consume more spores and so be more susceptible to infection, *Daphnia* that feed faster were, if anything, less likely to become infected in our study. Perhaps fast feeding is beneficial in some circumstances, despite the likely higher spore intake, because fast‐feeding *Daphnia* are able to use the extra resources collected by feeding quickly to fight infection. This result complements our finding that larger *Daphnia* are less likely to become infected: Both results seem to show that *Daphnia* who have access to more resources (either because they are larger at birth or feed faster) are better able to resist infection. The opposite expectation, that larger individuals feed faster, take up more spores, and are thus more susceptible to infection, has been used to explain why larger *D. dentifera* hosts are more likely to become infected with the fungus *M. bicuspidata* (Hall et al., 2007). This discrepancy might arise because the two studies looked at different life stages (adult *D. dentifera* and juvenile *D. magna*), although presently we do not have the data to test this.

Our aim in this study was to identify the mechanism by which low food triggers mothers to produce offspring that are more resistant to a bacterial pathogen (Ben‐Ami et al., 2010; Mitchell & Read, 2005; Stjernman & Little, 2011). Resistant offspring from low‐food mothers was the typical response across a large number of genotypes from a single *Daphnia* population, although not all genotypes respond identically (Stjernman & Little, 2011). In our study, we focused on a clone that displayed this average response of its population to understand the mechanism behind the maternal effect for the majority of clones in the population. The choice of a single clone is a compromise between measuring genetic diversity and gaining power to elucidate mechanism. It is of course of interest to speculate how body size will relate to infection risk for clones that do not show the average pattern and future experiments will expand upon the groundwork laid presently.

By showing that body size is an important determinant of susceptibility, our study also highlights a broad mechanism by which ecological and genetic factors can affect susceptibility and disease spread in populations. In addition to maternal food, *Daphnia* body size is determined by a number of factors (e.g., genetics, predator cues, clutch position), and these factors also have the potential to affect susceptibility through their effect on size. Body size effects might explain the variation in infection levels observed amongst *Daphnia* genotypes (Stjernman & Little, 2011) as well as providing a mechanistic link for phenomena such as the interplay between predator and parasite defense (Bertram, Pinkowski, Hall, Duffy, & Cáceres, 2013). Anything that changes the size‐structure of populations, like size‐selective predation (Galbraith, 1967; Gibson, 1980; Riessen & Young, 2005), also has the potential to influence disease resistance.

## Conflict of Interest

None declared.
